# Asian Community of Glycoscience and Glycotechnology (ACGG) conference report 2023

**DOI:** 10.1093/glycob/cwae017

**Published:** 2024-02-26

**Authors:** Kavita Y Hiremath

**Affiliations:** School of Life Science, Central University of Karnataka, Kadaganchi, Taluka, Aland District, Kalaburagi 585367, India

The ACGG Conference was initially started in Japan with a few Asian countries represented and has gradually gained in its strength by inclusion of other Asian countries. Presently the following countries are active members: China, Hongkong, India, Indonesia, Japan, Korea, Malaysia, Singapore, Taiwan and Thailand. Annual meetings are held in these Asian countries on a rotation basis and University of Hyderabad has earlier conducted the 6th ACGG meeting in December 2014. The conference included long and short talks as well as poster presentations on the broad topic of Glycobiochemistry.

Cancer is a leading disease worldwide, accounting for nearly 10 million deaths worldwide by 2020, which corresponds to one in 6 deaths. Most of the cancers can be cured by early diagnosis and appropriate treatment. Aberrant glycosylation on the cell surface is a hallmark of cancer. A common feature of cancer is the increased expression of N and O-linked glycosylation on cell surface glycoproteins. Cancer cells divide immortally by spreading cancer stem cells to other body parts. If aberrantly glycosylated glycans or biomarkers can be identified or diagnosed early, cancer patients can be treated with appropriate therapeutic agents. Current diagnostic implementations for cancer, like X-ray imaging, tomographic scan, sputum cytology, tissue removal, biopsy, etc., are costly, involve intense laboratory testing, or are less sensitive. Thus, there is a need to develop low-cost and sensitive methods to screen biomarkers, which require a small volume of readily available patient samples such as saliva, sputum, urine, etc. Therefore, important to discover novel techniques to identify biomarkers in disease-related conditions like cancer. To develop a consortium for delivering excellence in Glycobiology and Glycochemistry to identify cancer biomarkers for better healthcare, an international conference under the theme of Glycoscience and Glycochemistry was organized to disseminate information and to discuss ideas and controversial issues in the continuously evolving field of Glycobiochemistry.

About 150 participants from various educational research institutions in India and across Asian countries attended the conference. The Vice Chancellor of the University of Hyderabad Prof. B. Jagadeeshwar Rao inaugurated the conference, spoke about glycans as important cell surface components, and mentioned that the interaction of cells is mediated via the surface glycans and that they contain information as glyco-markers. He also emphasized that glycobiochemistry is one area with much to explore. Prof. N. Siva Kumar (Department of Biochemistry, School of Life Sciences) and Prof. Musti J. Swamy (School of Chemistry) University of Hyderabad welcomed all the conference delegates from across the Asian countries. Prof. Avadesha Surolia, a nationally and internationally distinguished glycobiologist attended the meeting and chaired a scientific session. They highlighted the scientific content of the conference.

Below is a summary of conference speaker topics.

- Atit Silsirivanit (Khon Kaen University, Thailand) spoke about how sialylation regulates stemness maintenance and drug resistance of cancer stem -like cells.
- N. C. Ramya (CSIR-Institute of Microbial Technology, Chandigarh, India) spoke about the potential of Maf glycosyltransferases to profoundly expand the scope of neo-glycopeptide synthesis and posttranslational protein engineering.
- Ali Rohman (Universitas Airlangga, Indonesia) spoke about engineered thermostable enzyme GH51 arabinofuranosidase activity, helpful in industrial applications.
- Shailaja Shefali (JNU, New Delhi, India) spoke about the characterized catalytic activity *of Candida albicans* GPi8 by designing different peptide substrates with altered residues.
- Madhulata (CSIR-IMTECH, Chandigarh, India) was used to identify four protein domains that bind to terminal N-glycans with fine variations in binding to human gut microbes.
- Jayaraman (IISc, Bangalore, India) discussed the synthesis of the smallest possible cyclic oligosaccharide that could be used in targeted drug delivery applications.
- Suvarn Kulkarni (IIT Bombay, Mumbai, India) spoke about rare or unique complex glycoconjugate synthesis and their applications in targeting bacterial surface glycan synthesis inhibition to stop their proliferation.
- Vinod Tiwari (Banaras Hindu University, Varanasi, India) discussed the synthesis of glycohybrids and glycodendrimers by using the CuAAC “Click” method, which has been recognized as a Nobel Prize-winning protocol, as well as being beneficial for drug delivery for chemotherapeutic purposes.
- Intzar Ali (UoH) discussed the synthesis of cardiac glycosides using the Mislow-Evans reaction.
- Atsushi Kuno (AIST, Japan) discussed constructing a strategic multimodal glyco-analysis system for therapeutic glyco-target research and development.
- Ni Nyoman Tri Puspaningsih (Universitas Airlangga, Indonesia) discussed the expression and characterization of a novel carbohydrate-binding module (CBM36) and its role in stabilizing lacaase.
- Lakshmi Sureka Krisnapati (UoH, India) discussed lysosomal L-fucosidase from the basal metazoan Hydra.
- Khoshya Neetu (CSIR-IMTECH, Chandigarh, India) examined the anti-biofilm effect of carbohydrate–active alginate lyase enzyme on *Pseudomonas aeruginosa*.
- Hiromune Ando (Gifu University, Japan) synthesized sialic acid- and KDO-containing glycans with controlled steric hindrance.
- Pooja Joshi (IISER Pune, India) spoke about synthesizing a library of glycolipids similar to the cell wall of *Mycobacterium tuberculosis*.
- Pavan K Kancherla (IIT Guwahati, India) discussed glycoside bond formation using strained and Brønsted pair catalysis methods.
- Chuan Fa Chang (National Cheng Kung University Taiwan) discussed how reduced sialylation promotes cancer stemness and metastasis in colorectal carcinoma.
- Sneha Sudha Komath (JNU, New Delhi, India) discussed the involvement of cross connections and biosynthesis of glycosyl phosphatidyl inositol (GPI) in the growth of the filamentous structure of *C. albicans*.
- Balakumaran Chandrasekar (BITS Pilani India) spoke about investigating the glycobiology of plant-microbe interactions.
- Shivaranjani (UoH, India) spoke about the physiological significance of lectins and glycosidases in bitter gourd and snake gourd, respectively.
- Sopit Wongkham (Khon Kaen University, Thailand) spoke about how a N-acetylgalactosaminyl transferase (GLNT 5) in promoting cholangiocarcinoma caused by liver fluke, which is most commonly found in Thailand and is overexpressed during the progression of cancer via the epidermal growth factor receptor (EGF-R) signalling pathway.
- Sutas Suttiprapa (Khon Kaen University Thailand) spoke about how a metallopeptidase enzyme derived from liver fluke that cause cholangiocarcinoma is crucial for host–parasite interactions.
- Monika Bharati (JNU New Delhi, India) discussed how GPI production in *C. albicans* is influenced by Arv1, an enzyme related to acyl-CoA acyltransferase essential for survival.
- Raghavendra Kikkeri (IISER Pune) spoke about heparin sulphate glycosylation of synthetic glycans to block heparinase and its antiviral properties.
- Srinivas Hota (IISER Pune) discussed utilizing the novel cut-insert-stitch editing reaction (CIStER) technology to edit oligosaccharides.
- Chebrolu Pulla Rao (SRM University, Guntur, India) discussed the computational docking of aromatic imino glycoconjugates to study carbohydrate-lectin/glycosidase interactions.
- Perali Ramu Sridhar (UoH, India) spoke about the production of carbon-branched sugars from glycans as vital bioactive natural compounds.
- Anne George (IIsc, Bangalore, India) discussed using anthracene as a probe to look at the bio-imaging capabilities of the recently produced anthracene methyl glycosides.
- Someswara Rao Sanapala (IIT Tirupathi, India) spoke about Pfizer sponsored effort for a semisynthetic glycoconjugate vaccine against antimicrobial-resistant *Escherichia coli* O25B.
- V. Kamala Lakshmi (UoH, India) discussed creating biologically active mixes of indole produced from carbohydrates by employing donor-acceptor cyclopropanes to create indole-oxepanone derivatives that function as a bridge.
- Vaishali Narayanan (IISc, Bangalore, India) spoke about Characterization of conformational fluctuation in non-mitogenic lectins.
- Appa Rao Podile (UoH, India) discussed plant immunity and the management of fungal diseases in relation to chitosans and chitooligosaccharides.
- Durba Roy (BITS-Pilani in Hyderabad, India) discussed using molecular dynamics to understand polysaccharides and polysaccharide-based membranes.
- Shaumashish Mukherjee (UoH, India) discussed how Paenibacillus sp. LS1’s multi-modular chitinase-5 breaks down crystalline chitin into N-acetyl-d-glucosamine and N, N-diacetyl chitobiose.
- M. Vandhana (UoH, India) discussed chitin’s molecular characterization and deconstruction by a CBM6 domain-containing chitinase from *Flavobacterium johnsoniae*.
- Kishore Babu Bobbili from Allahabad University in India talked about the interactions and structure of the cucumber phloem lectin Cus17.
- Patcharaporn Boottanun (AIST, Japan) discussed the enhancements to the LM-Glycome Atlas, a multimodality data visualization tool based on multi lectin specificity.
- Sneha Banerjee (UoH, India) spoke on the impact of crowding milieu on the T-antigen-specific lectin jacalin’s interaction with carbohydrate ligands and conformational changes in this protein.
- Ahallya Jaladeep (IISc, Bangalore, India) discussed about detecting ring-flipping dynamics via saturation transfer and relaxation dispersion NMR.

During the conference, after every talk, sufficient time was allocated for discussions with scientists from across Asian countries. Indian scientists have concluded the clinical applications of their respective research, and a few have helped to develop and identify cancer biomarkers and industrial applications of engineered enzymes and the prognosis of cancer.

Overall, the 12^th^ ACGG conference helped me to gain knowledge on importance of glycans in various biological processes and applications, including detecting cancer and host-pathogen interactions. The talks helped me to understand glycan screening, purification, analysis, synthesis, and determination of their structure, allowing modern researchers to design carbohydrate-based therapies with higher efficiency and a more effective selective disease-based fashion. Further knowledge of modified enzymes, their industrial applications, and how they are helpful for biodegradation is beneficial. In addition, conference talks highlighted research in glycobiochemistry to explore early diagnosis, prevention, and treatment of various diseases related to glycoscience and provide strong support for translational research and innovative approaches in developing medicines using glycobiochemistry knowledge.

The Conference activities were supported by generous funds from the Institute of Eminence at the University of Hyderabad.

